# Prediction of drowsiness using EEG signals in young Indonesian drivers

**DOI:** 10.1016/j.heliyon.2023.e19499

**Published:** 2023-09-03

**Authors:** Maya Arlini Puspasari, Danu Hadi Syaifullah, Billy Muhamad Iqbal, Valda Aqila Afranovka, Safa Talitha Madani, Armand Khalif Susetyo, Salsabila Annisa Arista

**Affiliations:** a Department of Industrial Engineering, Universitas Indonesia, Indonesia; b Centre for Business in Society, Coventry University, UK

**Keywords:** EEG signals, Drowsiness detection, Simulated driving, Sleep deprivation, Support vector machine

## Abstract

Indonesia is among the countries with the highest accident rates in the world. Fatigue and drowsiness are among the main causes of the increased risks of accidents in the road transport sector. Sleep-related factors (quality and quantity, time of day) and work-related factors significantly affect the development of fatigue. The EEG signal indicator is often referred to as the gold standard for measuring fatigue and drowsiness. However, previous studies focused primarily on the trends of EEG signals under certain conditions but overlooking the development of drowsiness indicators based on EEG signals. Furthermore, existing studies still do not agree on what parameters in the EEG signal indicator are best at detecting drowsiness. Thus, this study aims to design an EEG signal-based drowsiness indicator under simulated driving conditions. Drowsy drivers were monitored through EEG signal indicators and subjective assessments. The methods used in this study include statistical significance tests, logistic regression, and support vector machine. The results showed that sleep deprivation had a significant effect on increasing alpha, beta, and theta waves. In addition, driving duration significantly increased the theta power and all EEG ratios and decreased the beta power in the alert group. The ratio of (θ + α)/β and θ/β in the SD group also showed a considerable increase in the end of driving. Furthermore, sleep status and driving duration both influenced subjective sleepiness. EEG signals combined with sleep status and driving duration factors generated acceptable model accuracies (77.1% and 90.2% in training and testing, respectively), with 90.5% sensitivity and 90% specificity in data test. Support vector machine showed better classification than that of logistics regression, with the linear kernel as the best classifier. Theta power had the highest effect in the model compared with other EEG signals.

## 1 Introduction

Road accidents are among the most common causes of death in many countries. About 1.3 million people succumb to traffic accidents each year worldwide [1]. Traffic accidents rank tenth among the most common causes of death both in lower-middle- and upper-middle-income countries, according to 2019 accident data from the World Health Organization (WHO). In Indonesia, according to data from Statistics Indonesia, the number of accidents in 2019 reached more than 100,000 cases, with the death toll reaching more than 25,000 people. The annual growth of traffic accidents reaches 4.8% per year [2]. Meanwhile, according to data published by WHO in 2018, the number of deaths due to accidents in Indonesia reached 41,862 fatalities or 2.46% of all death cases. The potential socioeconomic losses calculated on the basis of the potential loss of income of accident victims reached more than Rp 250 billion per year [2]. In 2017, the number of accidents reached 104,000 cases, increased to 109,000 cases in 2018, and continued to increase to 116,000 cases in 2019. However, there was a decrease in the number of cases in 2020 to about 100,000 cases. Considering the situation, a decrease in the accident rate in 2020 may have occurred because of the Covid-19 pandemic, which caused most communities to reduce travel [3,4].

Three main factors cause road accidents: the physical factors of the road environment, vehicle factors, and human factors [5,6]. The physical factors of the road environment consist of poor road conditions (e.g., damaged roads, bumpy roads, sharp turns, traffic signs, and road markings), whereas vehicle factors include brakes not working properly, tires breaking on the road, and lights not working). Both physical factors of the road environment and vehicle factors can be classified as external factors, whereas human factors (in this case, the drivers) are classified as internal factors. Human factors are classified as internal because they involve the drivers and influence behaviors in driving, such as driving skills, personality, attitude, and fatigue [5,6].

Among the above three factors, about 90% of accidents are caused by human negligence factors [[5], [6], [7], [8], [9], [10]]. Al-Mekhlafi et al. [11] also argued that driver performance is critical in avoiding road crashes. According to data from the Indonesian National Police in 2017, 61% of the traffic accidents in Indonesia involved human factors related to the skill and character of drivers, 30% was caused by infrastructure and environmental factors, and the remaining 9% was caused by vehicle factors related to the fulfillment of engineering requirements.

Fatigue and drowsiness are one of the dominant human factors that cause accidents [12] Generally, the contribution of fatigue and drowsiness to road accidents is 20% of the overall mode of transport [13]. There are various causes of drivers' fatigue, such as circadian rhythm, sleep deprivation, duration of continuous driving, the drivers' personal characteristics, and driving environment [14]. According to previous studies, sleep-related factors (time of day and homeostatic factors) and work-related factors (duration of work, duration of rest, and work characteristics) are more dominant in influencing fatigue and drowsiness [15,16]. The closeness of the relationship between fatigue and drowsiness can reduce drivers' performance and decrease their ability to respond to or assess a condition such that inappropriate decision-making occurs, which can cause traffic accidents [15]. Meanwhile, Horrey et al. [17] stated that fatigue and drowsiness are considered the main causes of transportation accidents, which affect the magnitude of the mortality rate in traffic accidents. Further, work-related factors involve monotonous road conditions or high workload, traffic density, visibility, driving environment, and the presence of secondary tasks [16,18].

Drivers' sleepiness can be detected in several methods. Electrooculogram can measure the blink rate and duration of driving [19]. Drowsiness can also be captured from video images [20] or recorded through various analyses of electroencephalography [[21], [22], [23]]. EEG signals are considered the gold standard in measuring fatigue and drowsiness among other indicators, where there is an increase in alpha (*α*) and theta (*θ*) wave power and a decrease in beta wave power (*β*) in sleepy conditions [[24], [25], [26]]. In existing studies, there are numerous descriptions of EEG signal patterns as indicators of fatigue and drowsiness. According to the research conducted by Jagannath and Balasubramanian [27], there was an increase in *α* and *θ* waves along with an increase in driving duration and a significant decrease in *β* waves. Meanwhile, Jap et al. [26] examined several parameters of EEG signals, namely, *θ*/*β*, *θ*/(*α* + *β*), (*θ* + *α*)/*β*, and (*θ* + *α*)/(*α* + *β*), by driving on a train simulator for 30 min in monotony. They found that the parameter (*θ* + *α*)/*β* at the frontal and temporal points resulted in the most significant difference between alert and sleepy conditions. According to the research of Zuraida et al. [28], the increase in power *θ*, which is dominant in the frontal area, can be used as a strong indicator of fatigue.

Some previous research studies focused on classifying EEG signals into drowsy and alert conditions. For instance, Tuncer et al. [29] used EEG signals to classify fatigue using several machine learning methods, and they found *k*-nearest neighbors as a classifier with the highest accuracy. Meanwhile, other studies classified fatigue and alert conditions and found support vector machine (SVM) as the method with the highest accuracy compared with other methods [30]. Another research conducted by Zheng et al. [31] modified machine learning algorithms to detect fatigue. Through channel optimization and accuracy comparisons, the new feature selection approach had a good performance with the particle swarm optimization classifier [31]. Consistent with Barua et al. [30], a genetic-algorithm-based SVM can be used as a drowsiness classifier, with the gamma rhythm having the best detection efficiency of the five traditional rhythms [32].

From the results of the studies above, there are discrepancies on which parameters in brain signal indicators are best for detecting fatigue and sleepiness. There are also disagreements on the best classifier methods for detecting drowsiness. In recent years, several research works have focused on drowsiness detection while driving using EEG signal as an indicator. However, none of these studies have used the combinations of power-α, power-β, power-θ and four ratios of brainwaves (θ/β, θ/[α + β], [θ + α]/β, and [θ + α]/[α + β]) [33]. In addition, there is a need for detecting fatigue and drowsiness in real time while driving [34]. Another problem is the accuracy level of drowsiness classifications [35]. For these reasons, an EEG signal-based drowsiness indicator that can detect the onset of fatigue/drowsy states must be designed.

## 2 Methods

### 2.1 Participants

Thirty university students (18 males and 12 females) participated in this study (mean age: 20.6 ± 0.83 years). The participants were first screened out to ensure data homogeneity. There were several exclusion criteria for the participants. For instance, participants who were not heavy smokers (only smoked one to two cigarettes per day), were not caffeine addicts (maximum one cup per day) and did not consume drugs/alcohol were excluded from the study. Meanwhile, the inclusion criteria consisted of having experience in driving a car for at least a year, having normal eyes or normal eye correction, and being in good health condition. Those included also reported normal sleep durations (6 h/day on average) and the absence of sleep disturbance. This research involved students as participants by considering that young drivers are more likely to be involved in traffic incidents than older drivers [36].

The participants were also screened for their chronotype and daytime sleepiness level using the Morningness–Eveningness Questionnaire (MEQ) [37,38]. The chronotypes of the participants were tested using the MEQ to determine whether the subjects could be categorized as morning or evening people [39]. Extreme morning persons (MEQ score: 70–86) and extreme evening persons (MEQ score: 16–30) were excluded from this experiment [37]. Out of the 30 participants, four were of the moderate morning type, two were of the moderate evening type, and the rest were of the intermediate type.

The participants were asked to refrain from smoking and consuming all substances that could affect their fatigue level 24 h before the experiment, including coffee, tea, energy drinks, and medications [40,41]. Before the experiment began, all participants received detailed information about the experiment and gave written informed consent for their participation. The experimental procedures employed in this work complied to the ethical standards for using human subjects with the approval of The Research and Community Engagement Ethical Committee Faculty of Public Health Universitas Indonesia as a local ethics committee. The study complies with all regulations and informed consent was obtained from all participants, including the consent from the participants of their image to be published.

### 2.2 Experimental procedure

The participants in this study were divided into two groups: the alert group and the sleep-deprived (SD) group. In the alert group, the participants must sleep for at least 7 h the night before. In the SD group, the participants slept for less than 5 h. The alert group was utilized as a baseline for this study. The sleep conditions were measured using Fitbit Inspire 2, where the participants used such smartwatch 24 h before the experiment.

The participants familiarized the driving simulator before the session began. Before the experiment began, the blood pressures of the participants were recorded to ensure their fitness level. Personal data questionnaires and body mass indices were also asked of the participants. The participants were given lunch by the researchers before the driving session. They were instructed to drive simulated driving for 1 h with no specific scenario (free driving). The maximum speed required for each driver was 80 km/h. The traffic was set to 70% to simulate Jakarta's traffic condition.

In this study, the participants were asked to drive in the simulator for 1 h in the time range of 13:00–15:00. The total duration of data collection for each respondent was approximately 85 min, consisting of 5 min for participants to fill in personal data, measure body mass index, and blood pressure, 15 min to conduct briefings related to the experiments and familiarization with the driving simulators, 5 min for EEG installation, and 60 min to conduct data collection. In addition, the respondent's Karolinska Sleepiness Scale (KSS) scores were recorded every 15 min. Table 1 shows the details of the experimental procedure.

**Table 1:** Experimental procedure.

5 min	15 min	5 min	60 min	Total time
Filling in the questionnaire, weight and blood pressure measurements	Briefing on the experiment and familiarization with the driving simulator	EEG installation	Data gathering using the driving simulator EEG data were recorded for the entire 60 min KSS scores were asked every 15 min	85 min

The participants were asked to control their sleep duration the day before the experiment using an activity tracker named Fitbit Inspire 2. If the participants' sleep duration was not in the range of 4.5–5 h (in the SD group condition) and 7–8 h (in the alert group), the participants were asked to redo the experiment on another day. Because of the little control over the participants' sleep duration (only relying on the Fitbit device), the actual sleep duration of the participants varied from 4.6 ± 0.79 h (for the SD group) to 7.19 ± 0.44 h (for the normal sleep condition).

### 2.3 Data recording

**Fig. 1:**
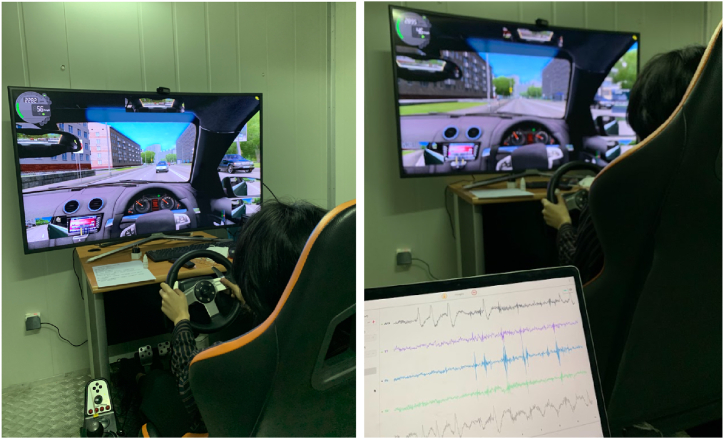
Data collection from the experiment.

A medium fidelity driving simulator was employed in this investigation. Three-foot pedals, a force-feedback steering wheel, and a gear shift lever were all part of the simulator (G27 92, Logitech, USA). The driving surroundings were projected onto a 55-inch HD screen to provide visual input. Driving simulator software (City Car Driving version 1.4, Multisoft, Russia) was used in this study (Fig. 1). A highway with medium traffic density (70% traffic), typical daylight hours, and normal traffic behavior was chosen as the driving environment for this investigation. A video camera was used to record the experiment and monitored by an experimenter sitting in the same room. Throughout the experiment, the brainwaves were monitored and recorded using an EEG (Emotiv Insight, USA).

### 2.4 EEG signals

EEG signals were measured using Emotiv Insight. The device used a five-channel noninvasive Bluetooth-enabled device, with fewer points of contact than existing EEG devices and the capacity to wirelessly acquire EEG brain data [42]. The EEG signals were recorded throughout the sessions to reduce task interruption due to the EEG installation process. The EEG data were later grouped into 15-min durations.

The eeglab module in MATLAB was used to extract the signals (stored in edf files). Each EEG wave underwent band-pass filtering to remove unwanted signal frequencies between 0.5 and 32 Hz [43]. The power spectral density (PSD) values of the theta waves (4–8 Hz), alpha waves (8–13 Hz), and beta waves (13–25 Hz) were obtained using signal decomposition and then converted to dB units [44]. The mean value of the 15-min PSDs was used to represent each brain wave as an EEG metric. The EEG parameters of power-*α*, power-*β*, power-*θ* and four ratios of brainwaves (*θ*/*β*, *θ*/[*α* + *β*], [*θ* + *α*]/*β*, and [*θ* + *α*]/[*α* + *β*]) were analyzed [26,28]. The EEG parameters were selected based on the significant difference found for the four ratios of brainwaves between alert and fatigue in driving conditions; these four ratios of brainwaves were considered to be good indicators of driver drowsiness [26,45,46]. This study also excluded delta and gamma waves because they were not relevant to the research on drowsiness detection. In particular, delta waves frequently occur during deep sleep state [47,48], while gamma waves are related to excitement feeling and higher brain functions, such as learning, memory, and information processing [33,49].

### 2.5 Subjective sleepiness

Subjective alertness was assessed using the KSS [50]. The KSS is a single-item scale that allows participants to rate their current sleepiness on a 9-point scale (1 = extremely alert, 9 = very sleepy, fighting sleep, difficulty staying awake). The KSS used in this study was translated to Indonesian to avoid bias during the assessment. Every 15 min, the participants were asked to rate their subjective sleepiness using the KSS questionnaire. The 15-min time frame was determined as the KSS measurement should not be below the 15-min interval as it would affect the alertness of the participants [51].

### 2.6 Data analysis

The data for each participant were aggregated over a time window of 15 min and were shown as mean and standard deviation. Data outliers were eliminated using Tukey's box plot [52]. The collected data were checked for normal distribution using the Kolmogorov–Smirnov test. The data were also transformed using the log10 function. Except for KSS, all data were normally distributed. One-way analysis of variance (ANOVA) with the alert/SD group (normal sleep vs sleep-deprived) was performed to analyze the EEG signals, and the Mann–Whitney *U* test was used to assess KSS. Repeated measure ANOVA was employed to assess the effect of driving duration on the EEG signals, and the Friedman test was used to assess the effect of driving duration on KSS. These tests were used to determine whether there were any statistically significant differences between the means of the groups. Reportedly, the data from one participant in the SD group were outliers and were therefore excluded from the analysis.

**Fig. 2:**
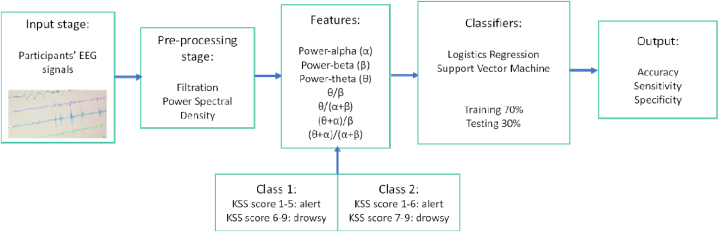
Data classification process.

The data were then classified into alert and drowsy conditions according to the KSS score. In this research, we compared two classifications based on the KSS score, because a review of previous literature reveals diverse opinions regarding the measurement of the onset of sleepiness. The first classification was KSS 1–5, categorized as “alert,” whereas KSS 6–9 was categorized as “drowsy” (Fig. 2). The classification was based on the study of Barua et al. [30], which stated that “somewhat sleepy” drivers fall into the category of KSS 6–7. The second classification was based on Wang and Xu [53], who stated that the cutoff of the drowsy condition is at KSS 7. The use of subjective sleepiness ratings as the ground truth for sleepiness was motivated by the fact that KSS is simple to be applied, inconspicuous, and commonly used in driver-sleepiness studies [30,54]. Binary logistics regression and SVM were performed to classify the alert and drowsy states. Then, 70% of the data was trained, whereas the rest was used as the test dataset [30,54]. The analysis associated with building the logistic regression and SVM model was calculated using MATLAB R2022a.

The performances of the classifiers considered in this research were accuracy, sensitivity, and specificity. Accuracy gauged how well the model to correctly predict every sample. Sensitivity and specificity measured the ability of the model to correctly predict the drowsy and the normal states, respectively. True positive (TP) was the number of drowsy states classified as drowsy states, and true negative (TN) was the number of normal states identified as normal states. Meanwhile, false positive (FP) was the number of normal driving states classified as drowsy states, and false negative (FN) was the number of drowsy states identified as normal driving states. The following are the equations for the performance measurement parameters:

(1) $$\text{Accuracy}=\frac{TP+TN}{TP+TN+FP+FN}\times 100\%$$

(2) $$\text{Sensitivity}=\frac{TP}{TP+FN}\times 100\%$$

(3) $$\text{Specificity}=\frac{TN}{TN+FP}\times 100\%$$

In this study, ANOVA as the feature selection method were used based on the continuous predictors in the model [55] so as to eliminate unnecessary and redundant features that do not contribute to the model performance [56]. In addition, principal component analysis (PCA) was used as the feature transformation technique that transforms existing features into new features [57]. This study also compared the results of the classifier with PCA and without PCA (no feature transformation) to obtain the highest accuracy and lowest computation time.

The G-power 3.1 software was used to determine whether the study's sample size was sufficient [58]. A sample of 15 participants produced a statistical power (1−β) of 0.82, which was higher than the recommended minimum statistical power of 0.8, according to the estimated statistical power analysis results [59].

## 3 Results

### 3.1 Subjective sleepiness

The subjective sleepiness level as measured by KSS was significantly impacted by sleep deprivation (*p* < 0.001). The participants in the SD group reported higher subjective sleepiness scores (KSS score = 5.93 ± 1.95) than those in the alert group (KSS score = 4.83 ± 1.46).

**Fig. 3:**
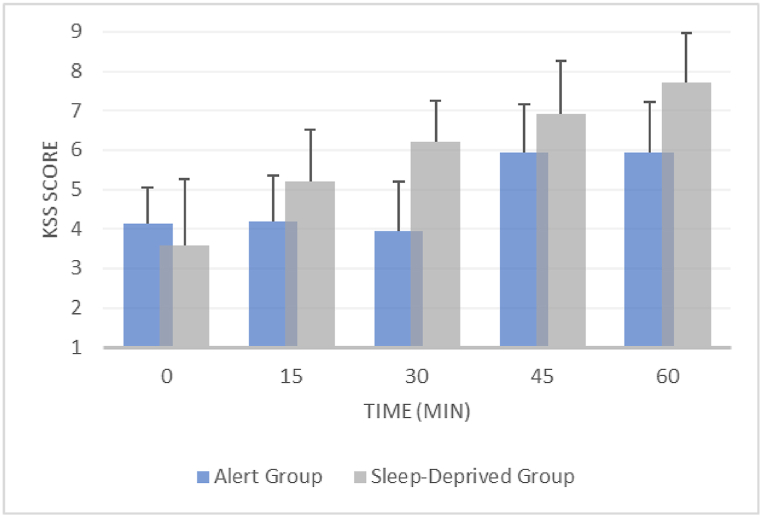
Trends of KSS scores over 1 h of driving.

The effect of driving duration was significant toward subjective sleepiness in the SD group (χ2(4) = 42.950, *p* < 0.001) and in the alert group (χ2(4) = 34.868, *p* < 0.001). The subjective sleepiness slowly increased as the driving duration reached 1 h (Fig. 3).

### 3.2 EEG signals

**Fig. 4:**
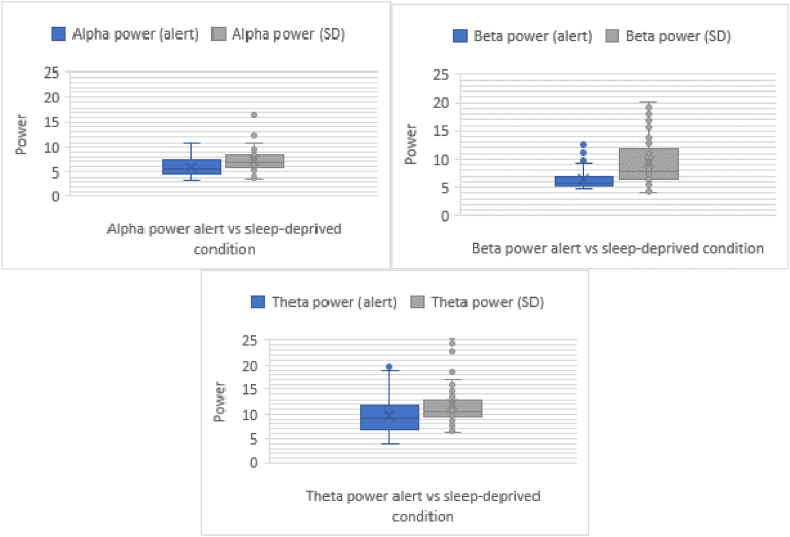
Effects of alert and SD group towards the power of *α*, *β*, and *θ* waves.

The summary of the EEG parameters was shown in Table 2. As reported, the *α*, *β*, and *θ* waves were closely associated with the driver fatigue state [26]. The intergroup comparison demonstrated that the *α* power between the two groups (alert and SD groups) was significant (*F* (1,28) = 22.957, *p* < 0.001). The participants in the alert group had significantly lower *β* power (*F* (1,28) = 38.485, *p* < 0.001) and *θ* power than those in the SD group (*F* (1,28) = 12.414, *p* < 0.001), as shown in Fig. 4. In contrast, all the ratios did not exhibit a difference between the alert and SD groups (Table 3).

**Table 2:** Summary of the parameters of EEG signals.

Group	Driving Duration (mins)				
	0	15	30	45	60
**Alert Group**					
*α*	5.71 ± 1.43	6.19 ± 1.70	5.86 ± 2.07	5.38 ± 1.68	6.22 ± 2.03
***β***	6.23 ± 1.55	6.54 ± 1.77	7.23 ± 2.52	5.89 ± 1.14	5.85 ± 0.67
***θ***	9.75 ± 4.34	9.46 ± 3.59	8.32 ± 2.14	9.37 ± 3.26	11.93 ± 4.61
***(θ + α)/β***	2.54 ± 0.77	2.34 ± 0.38	2.02 ± 0.45	2.49 ± 0.55	3.12 ± 0.87
***θ/β***	1.60 ± 0.71	1.36 ± 0.35	1.22 ± 0.33	1.59 ± 0.51	2.08 ± 0.84
***θ/(α + β)***	0.83 ± 0.35	0.69 ± 0.18	0.67 ± 1.55	0.84 ± 0.26	1.02 ± 0.41
***(θ + α)/(α + β)***	1.30 ± 0.34	1.18 ± 0.17	1.11 ± 0.18	1.31 ± 0.26	1.53 ± 0.41
**Fatigue Group**					
*α*	7.63 ± 1.94	6.80 ± 2.22	6.97 ± 1.81	7.53 ± 1.73	7.70 ± 3.16
***β***	10.42 ± 4.04	9.85 ± 5.18	8.59 ± 3.90	8.50 ± 2.68	9.47 ± 5.17
***θ***	12.73 ± 4.96	11.69 ± 5.72	11.79 ± 4.57	13.28 ± 6.17	10.41 ± 2.38
***(θ*** + ***α)/β***	2.19 ± 0.95	2.16 ± 0.91	2.42 ± 0.92	2.64 ± 0.85	2.47 ± 0.90
***θ/β***	1.39 ± 0.74	1.38 ± 0.69	1.55 ± 0.70	1.72 ± 0.68	1.54 ± 0.67
***θ/(α*** + ***β)***	0.74 ± 0.31	0.74 ± 0.29	0.80 ± 0.29	0.87 ± 0.30	0.77 ± 0.27
***(θ*** + ***α)/(α + β)***	1.18 ± 0.37	1.16 ± 0.36	1.25 ± 0.37	1.34 ± 0.34	1.24 ± 0.34

**Table 3:** Summary of the effects of the alert/SD group.

Parameters	*F*-value
*α*	22.957**
*β*	38.485**
*θ*	12.414**
(*θ* + *α*)/*β*	2.159
*θ*/*β*	0.894
*θ*/(*α* + *β*)	2.035
(*θ* + *α*)/(*α* + *β*)	2.401

**p < 0.010.

*p < 0.050.

**Fig. 5:**
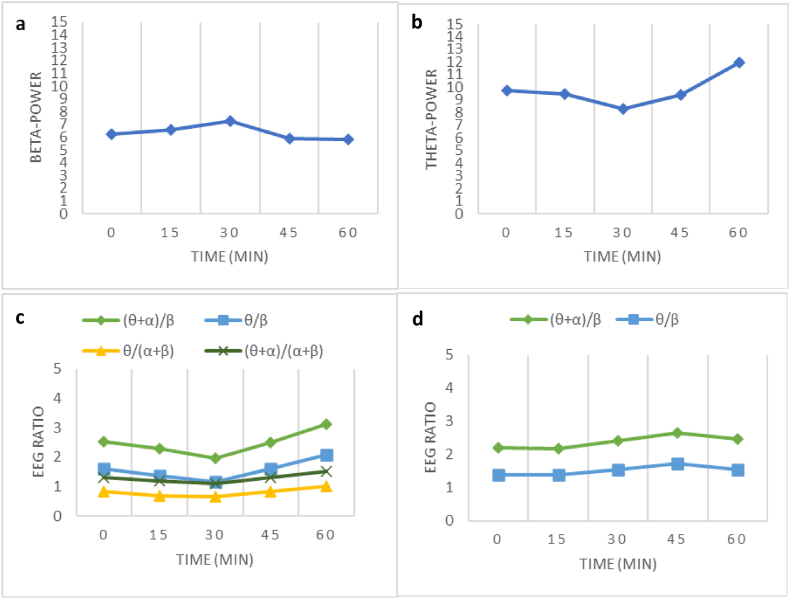
Effects of driving duration on the EEG signals. (**a**) effect of driving duration towards power of *β* in alert group, (**b**) effect of driving duration towards power of *θ* in alert group, (**c**) effect of driving duration towards the EEG ratio in alert group, (**d**) effect of driving duration towards the EEG ratio in SD group.

The effects of driving duration on the EEG signals varied. In the alert group, the *β* wave (*F* (4,56) = 3.337, *p* < 0.050) showed a significant decrease at the end of driving. Furthermore, the *θ* wave (*F* (4,56) = 3.106, *p* < 0.050) exhibited a significant increase at the end of the driving session. All EEG ratios also exhibited a significant change in driving duration, where all ratios increased at the end of driving (Fig. 5). In the SD group, the driving duration exhibited a significant increase at the end of driving, as shown by the ratio (*θ* + *α*)/*β* and *θ*/*β* (Table 4). It showed that the participants already experienced fatigue at the beginning of the driving session, and there was a slight increase in drowsiness by the end of driving.

**Table 4:** Summary of the effects of driving duration.

Parameters	Alert group (*F*-value)	SD group (*F*-value)
*α*	1.660	1.110
*β*	3.337*	0.427
*θ*	3.106*	0.829
(*θ* + *α*)/*β*	6.652**	3.324*
*θ*/*β*	5.466**	2.934*
*θ*/(*α* + *β*)	4.609**	2.238
(*θ* + *α*)/(*α* + *β*)	5.271**	2.554

**p < 0.010; *p < 0.050.

### 3.3 Drowsiness classification

The statistical significance of each EEG variable was evaluated, and the nonsignificant variables were removed. All EEG characteristics among the subjects of the study were identified to be significant as determined by the 95% credible interval. After the elimination of outliers, 137 data were run in the model. All variables of the EEG signals were included in the model. The predictors consisted of power-*α*, power-*β*, power-*θ*, *θ*/*β*, *θ*/(*α* + *β*), (*θ* + *α*)/*β*, (*θ* + *α*)/(*α* + *β*). The classifiers used in the model were logistics regression and SVM. SVM models were also divided into linear SVM, quadratic SVM, cubic SVM, and Gaussian-based to further compare and obtain the highest accuracy of the model. We conducted oversampling by adding the copy of the data categorized as “drowsy” for class 2 because of the imbalanced data.

The results of the analysis showed that the fine gaussian kernel classifier generated the highest accuracy for both classes 1 and 2 (Table 5). These are similar to the findings of a previous study, where the SVM showed the best performance among other classifiers [30]. In class 1, the accuracy of the training and testing data reached 59.4% and 63.4%, respectively. Meanwhile, class 2 has higher accuracy, with 72.9% accuracy in training and 78% accuracy in testing. However, the specificity of the model was very low (3.8% and 10% in training and testing, respectively).

**Table 5:** Model classification accuracy.

Class	Feature transformation	Classifier	Training accuracy	Testing accuracy	Sensitivity (training)	Specificity (training)	Sensitivity (testing)	Specificity (testing)
1 (cutoff = KSS 6)	Principal Component Analysis	Logistics regression	53.10%	58.50%	73.10%	29.50%	90.50%	25.00%
		SVM (Linear)	52.10%	51.20%	76.90%	22.70%	90.50%	10.00%
		SVM (Quadratic)	52.10%	53.70%	76.90%	22.70%	100.00%	5.00%
		SVM (Cubic)	49.00%	43.90%	65.40%	29.50%	66.70%	20.00%
		SVM (Fine gaussian)	57.30%	53.70%	75.00%	36.40%	57.10%	50.00%
		SVM (Medium gaussian)	57.30%	51.20%	94.20%	13.60%	95.20%	5.00%
		SVM (Coarse gaussian)	54.20%	51.20%	100.00%	0.00%	100.00%	0.00%
	No Feature Transformation	Logistics regression	49.00%	51.20%	59.60%	36.40%	81.00%	20.00%
		SVM (Linear)	45.80%	48.80%	69.20%	18.20%	90.50%	5.00%
		SVM (Quadratic)	44.80%	51.20%	59.60%	27.30%	71.40%	30.00%
		SVM (Cubic)	55.20%	65.90%	69.20%	38.60%	61.90%	70.00%
		SVM (Fine gaussian)	59.40%	63.40%	65.40%	52.30%	61.90%	65.00%
		SVM (Medium gaussian)	45.80%	41.50%	71.20%	15.90%	76.20%	5.00%
		SVM (Coarse gaussian)	55.20%	53.70%	94.20%	9.10%	100.00%	5.00%
2 (cutoff = KSS 7)	Principal Component Analysis	Logistics regression	70.80%	70.70%	94.30%	7.70%	87.10%	20.00%
		SVM (Linear)	70.80%	75.60%	97.10%	0.00%	100.00%	0.00%
		SVM (Quadratic)	70.80%	73.20%	97.10%	0.00%	96.80%	0.00%
		SVM (Cubic)	68.80%	78.00%	85.70%	23.10%	83.90%	60.00%
		SVM (Fine gaussian)	72.90%	78.00%	98.60%	3.80%	100.00%	10.00%
		SVM (Medium gaussian)	71.90%	75.60%	98.60%	0.00%	100.00%	0.00%
		SVM (Coarse gaussian)	72.90%	75.60%	100.00%	0.00%	100.00%	0.00%
	No Feature Transformation	Logistics regression	68.80%	68.30%	91.40%	7.70%	80.60%	30.00%
		SVM (Linear)	69.80%	75.60%	95.70%	0.00%	100.00%	0.00%
		SVM (Quadratic)	68.80%	73.20%	92.90%	3.80%	96.80%	0.00%
		SVM (Cubic)	62.50%	75.60%	78.60%	19.20%	87.10%	40.00%
		SVM (Fine gaussian)	72.90%	75.60%	97.10%	7.70%	96.80%	10.00%
		SVM (Medium gaussian)	71.90%	75.60%	98.60%	0.00%	98.60%	0.00%
		SVM (Coarse gaussian)	72.90%	75.60%	100.00%	0.00%	100.00%	0.00%

Furthermore, the classification model was improved by adding two internal factors of the participants as features, due to the problem with specificity. The alert and drowsy status of the participants were recorded based on their group. Alert participants slept for more than 7 h in the previous night, while drowsy participants slept less than 5 h in the previous night. Driving time was classified based on the sequence of driving, where each participant has five sequences: 0 min as the beginning of driving, 15 min after driving, 30 min after driving, 45 min after driving, and 60 min after driving that mark as the end of driving tasks. The summary of the revised classification is shown in Table 6. Overall, the accuracy, sensitivity, and specificity of the models were improved compared with those of previous models. In both classifications, the SVM model overall performed better than did the logistic regression model in terms of the training and testing overall accuracy. In class 1, SVM in the linear kernel had the highest accuracy in training and testing (77.1% and 90.2%, respectively), with 90.5% sensitivity and 90% specificity in testing. In class 2, SVM in linear kernel performed better than did the other classifier with 82.3% accuracy in training and 85.4% accuracy in testing. Overall, class 1 with KSS 6 as the cutoff value performed better in terms of testing accuracy than did class 2. The model that did not utilize PCA has the highest accuracy compared to the model using PCA.

**Table 6:** Model classification accuracy with added features.

Class	Feature transformation	Classifier	Training accuracy	Testing accuracy	Sensitivity (training)	Specificity (training)	Sensitivity (testing)	Specificity (testing)
1 (cutoff = KSS 6)	Principal Component Analysis	Logistics regression	71.90%	78.00%	76.90%	65.90%	76.20%	80.00%
		SVM (Linear)	74.00%	87.80%	75.00%	72.70%	81.00%	95.00%
		SVM (Quadratic)	74.00%	87.80%	82.70%	63.60%	85.70%	90.00%
		SVM (Cubic)	68.80%	82.90%	80.80%	54.50%	81.00%	85.00%
		SVM (Fine gaussian)	77.10%	82.90%	73.10%	81.80%	85.70%	80.00%
		SVM (Medium gaussian)	76.00%	87.80%	78.80%	72.70%	85.70%	90.00%
		SVM (Coarse gaussian)	61.50%	78.00%	92.30%	25.00%	95.20%	60.00%
	No Feature Transformation	Logistics regression	70.80%	82.90%	73.10%	68.20%	85.70%	80.00%
		SVM (Linear)	77.10%	90.20%	73.10%	81.80%	90.50%	90.00%
		SVM (Quadratic)	72.90%	80.50%	76.90%	68.20%	85.70%	75.00%
		SVM (Cubic)	65.60%	68.30%	65.40%	65.90%	71.40%	65.00%
		SVM (Fine gaussian)	61.50%	70.70%	71.20%	50.00%	85.70%	55.00%
		SVM (Medium gaussian)	65.60%	85.40%	69.20%	61.40%	90.50%	80.00%
		SVM (Coarse gaussian)	63.50%	75.60%	88.50%	34.10%	95.20%	55.00%
2 (cutoff = KSS 7)	Principal Component Analysis	Logistics regression	80.20%	80.50%	85.90%	64.00%	96.70%	36.40%
		SVM (Linear)	83.30%	80.50%	93.00%	56.00%	96.70%	36.40%
		SVM (Quadratic)	79.20%	80.50%	87.30%	56.00%	96.70%	36.40%
		SVM (Cubic)	78.10%	78.00%	87.30%	52.00%	96.70%	27.30%
		SVM (Fine gaussian)	77.10%	78.00%	90.10%	40.00%	96.70%	27.30%
		SVM (Medium gaussian)	82.30%	80.50%	93.00%	52.00%	96.70%	36.40%
		SVM (Coarse gaussian)	74.00%	73.20%	100.00%	0.00%	100.00%	0.00%
	No Feature Transformation	Logistics regression	78.10%	78.00%	84.50%	60.00%	90.00%	45.50%
		SVM (Linear)	82.30%	85.40%	91.50%	56.00%	100.00%	45.50%
		SVM (Quadratic)	80.20%	80.50%	85.90%	64.00%	90.00%	54.50%
		SVM (Cubic)	76.00%	75.60%	81.70%	60.00%	83.30%	54.50%
		SVM (Fine gaussian)	76.00%	73.20%	97.20%	16.00%	100.00%	0.00%
		SVM (Medium gaussian)	80.20%	75.60%	94.40%	40.00%	96.70%	18.20%
		SVM (Coarse gaussian)	74.00%	73.20%	100.00%	0.00%	100.00%	0.00%

Further analysis using ANOVA as the feature selection found that the *θ* wave had the highest-ranked features compared with those of the other EEG variables. *θ*/(*α* + *β*) and (*θ* + *α*)/(*α* + *β*) were the second and third in the feature rank order. This agrees with the results of the EEG signals significance analysis, signifying the importance of the *θ* wave in determining drowsiness onset.

**Fig. 6:**
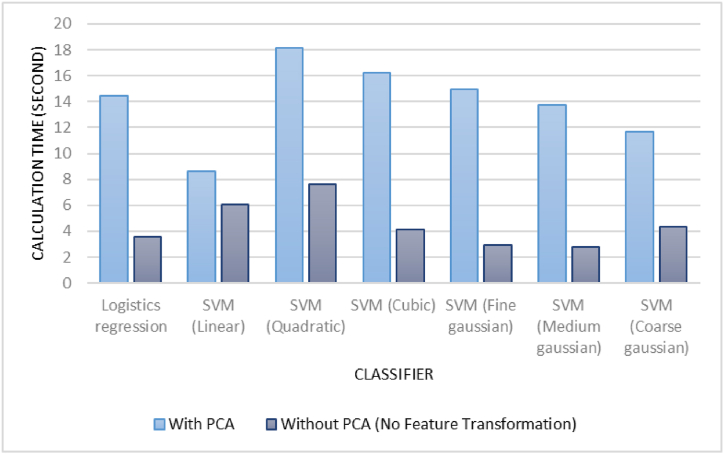
Computation time.

Fig. 6 shows the computation time decreased with the removal of PCA. In addition, the training time of selected classifier (linear SVM) was found to be 6.0581 s and the prediction speed was 1900 observation/second. This finding confirms the feasibility of the classifier for real-time detection when driving. In the future research, an alarm system containing the visual and auditory countermeasures can be developed to detect driver drowsiness directly [15].

## 4 Discussion

### 4.1 Trend of EEG signals and drowsiness detection

From the significance analysis, we conclude that the results of the EEG signals were varied. Sleep deprivation (SD group) caused an increase in the *α*, *β*, and *θ* power compared with the normal sleep condition (alert group). When exhausted, a driver's *θ* and *α* power are significantly higher than those when non-fatigued. As Zuraida et al. [28] noted, the increase in the *θ* wave is more dominant than those of the others. Meanwhile, other studies demonstrated that deteriorating performance and diminished alertness are accompanied by an increase in *θ* and a decrease in *β* activity [14,60]. The study of Jap et al. [26] demonstrated that throughout the repeated transitional phase from alertness to drowsiness, *θ* activity rises as *β* activity falls; however, at the start of the trial, *α* activity was constant, and it only started to decline near the end of the driving period.

There were some interesting findings related to the driving duration factor. Driving duration affected the alert group, where the *θ* power and the entire EEG ratio showed significant increases at the end of driving, whereas the *β* power decreased significantly at the end of driving. However, in the SD group, two ratios had a slight effect on the driving duration, which implies that the participants were fatigued at the early stage of driving. Moreover, between time periods, the EEG parameters sometimes increased, but they decreased in other periods of time. Their changes fluctuated and were not linear. These results are aligned with previous research by Zuraida et al. [28], where brain wave signals did not show consistent changes as the driving duration increased. However, a study of 1-h driving tasks performed in the field conducted by Perrier et al. [61] showed pronounced changes in *θ* and *α* wave activities, along with a more subtle increase in *β* activities. Compared with this study, the result for the *β* wave in the alert group showed a significant decrease at the end of driving, which was also in contrast with previous study results.

The participants' KSS scores indicated a decline in alertness and an increase in fatigue, as shown by the progressive increase in sleepiness ratings. Similar to the results of the previous study by Zuraida et al. [28], the subjective rating toward sleepiness in the SD group was significantly higher than that of the alert group. The results also show that the driving duration significantly affected both the alert group and the SD group. There is dissimilarity between the EEG signals and the subjective sleepiness results. In the SD group, sleepiness increased as the duration of driving increased, whereas the EEG signals did not change with the increase in driving duration. This shows the difference between the EEG signals and subjective sleepiness, which affected the drowsiness detection model. This is an indicator that KSS may be less reliable in detecting subjective sleepiness because participants feel biased when answering questions about their sleepiness levels. According to the study of Wijayanto et al. [62], there is another influential factor that is affected by sleep deprivation: situational awareness. Situation awareness becomes the mediating factor between sleep deprivation and driving performance. In future research, the effect of situational awareness can be further investigated.

From the evaluations, we observed that the EEG signals were reliable factors when used to determine the driver's drowsiness state. The results of the classification show that other internal factors (e.g., sleep status and duration of driving) added to the model significantly increased the classification accuracy. This is in line with the study of Liang et al. [63], which stated that the internal factors of participants improved the prediction accuracy of classification models. This result is also in line with the finding that creating personalized models for each driver was a crucial component in building predictive models for driver cognitive distraction [63]. This result is not unexpected, given the vast range of inter-individual variations in sleepiness, chronic and acute sleep effects, chronotype, and many other factors [15,64].

The overall performance of these models was promising (class 1 model testing accuracy 90.2%; class 2 model testing accuracy 85.4%). Class 1 is preferable to class 2 for drowsiness detection because of its higher accuracy, sensitivity, and specificity in the testing data. This model generated a slightly higher sensitivity than specificity. According to Akerstedt et al. [41], in real driving, a higher level of specificity is preferred to minimize false alarms. The higher level of specificity also increases drivers' confidence in the warning system [41]. In-depth research is required to determine the degree of sensitivity and specificity that practitioners in actual driving situations will tolerate.

In the results, *θ* power had the highest feature importance compared with those of the other EEG parameters. This result agrees with Jap et al. [26] and Barua et al. [30], who stated that the dominant characteristics is the energy ratio between lower frequencies (*α* and *θ*, indicating drowsiness) and higher frequencies (especially *β*, indicating alertness). The dominance of the *θ* wave is also similar to that in the study of Zuraida et al. [28], which stated the increase in the *θ* wave was more dominant than those of others.

The SVM classifier showed the highest accuracy results compared with logistics regression. This is in line with the study of Barua et al. [30] and Wang et al. [32]. Compared with logistics regression, SVM identifies the hyperplane that maximizes the geometric margin in the classification while simultaneously minimizing the empirical classification error [65]. The classification problem becomes straightforward in this feature space because SVM can convert the original data points into a high-dimensional feature space. Consequently, classification issues involving redundant data sets can be handled by the SVM [66]. This study found that SVM with a linear kernel is superior to other SVM types (e.g., Gaussian-based and non-linear). This study also found that the removal of PCA increased the accuracy and decreased the computation time. The findings are consistent with Yu et al. [57], that claimed when the feature dimension is more than the sample size, PCA is not appropriate. Additionally, feature extraction may lose interpretability and easily result in data information loss [57].

### 4.2 Limitations and future work

This study highlights some limitations into account. First, a medium fidelity driving simulator was used in this investigation. On the basis of driving behavior, Meuleners and Fraser [67] found that the results from a driving simulator were not significantly different from those of real driving (e.g., compliance with traffic signage and maximum driving speed). Additionally, Davenne et al. [68] claimed that, aside from how well drivers performed, subjective sleepiness did not change much between real-world driving versus driving in a simulator, except for the performance of drivers. However, in the current study, we discovered an increase in drowsiness while driving during daylight without experiencing sleep deprivation, showing the impacts of the simulator itself [41]. Thus, actual driving should be included in future studies to enhance the current study's validity. Second, this study used a small sample size. More research with a bigger sample size is therefore desired to improve the validity of EEG signals as drowsiness detectors.

## 5 Conclusions

This study designed an EEG signal-based drowsiness indicator in simulated driving conditions. The results showed that sleep deprivation significantly affects the increase in *α*, *β*, and *θ* waves. In addition, driving duration significantly increased the *θ* power and all EEG ratios and decreased the *β* power in the alert group. Furthermore, sleep status and driving duration both influenced subjective sleepiness. The EEG signals combined with sleep status and driving duration factors generated a model accuracy of 77.1% in data training and 90.2% in data testing with 90.5% sensitivity and 90% specificity. The SVM showed better classification than that of logistic regression, with the linear kernel as the best classifier. *θ* power had the highest effect in the model compared with other EEG signals.

## Declaration of competing interest

The authors declare that they have no known competing financial interests or personal relationships that could have appeared to influence the work reported in this paper.

## References

[1] World Health Organization (2018). Road traffic injuries. http://www.who.int/mediacentre/factsheets/fs358/en/.

[2] Statistics Indonesia (2020). Jumlah kecelakaan, korban mati, luka berat, luka ringan, dan kerugian materi yang diderita tahun 2017-2019. https://www.bps.go.id/indicator/17/513/1/jumlah-kecelakaan-korban-mati-luka-berat-luka-ringan-dan-kerugian-materi.html.

[3] Alfarizi M. (2021). Jumlah kecelakaan 2020 turun 14 persen karena pandemi. *Tempo*. https://otomotif.tempo.co/read/1447846/jumlah-kecelakaan-2020-turun-14-persen-karena-pandemi.

[4] Wu C.H., Kao S.C., Chang C.C. (2020). A knowledge elicitation approach to traffic accident analysis in open data: comparing periods before and after the Covid-19 outbreak. Heliyon.

[5] Ulleberg P R.T. (2003). Personality, attitudes and risk perception as predictors of risky driving behaviour among young drivers. Saf. Sci..

[6] Shope J.T. (2006). Influences on youthful driving behavior and their potential for guiding interventions to reduce crashes. Inj. Prev..

[7] Nzuchi J.S., Ngoma S.J., Meshi E.B. (2022). Commercial motorcyclists and road safety measures compliance. A case study of Dodoma city, central Tanzania. Heliyon.

[8] Hole A.R. (2007). Fitting mixed logit models by using maximum simulated likelihood. STATA J.: Promoting Communications on Statistics and Stata.

[9] S D. (1978).

[10] Yilmaz V., Çelik H.E. (2004). A model for risky driving attitudes in Turkey. SBP (Soc. Behav. Pers.): Int. J..

[11] Al-Mekhlafi A.A., Isha A.S.N., Abdulrab M., Ajmal M., Kanwal N. (2022). Moderating effect of safety culture on the association inter work schedule and driving performance using the theory of situation awareness. Heliyon.

[12] Okafor S., Adanu E.K., Jones S. (2022). Severity analysis of crashes involving in-state and out-of-state large truck drivers in Alabama: a random parameter multinomial logit model with heterogeneity in means and variances. Heliyon.

[13] Zhang G., Yau K., Chen G. (2013). Risk factors associated with traffic violations and accident severity in China. Accid. Anal. Prev..

[14] Eoh H., Chung M., Kim S. (2005). Electroencephalographic study of drowsiness in simulated driving with sleep deprivation. Int. J. Ind. Ergon..

[15] Williamson A., A L.D., Folkard S., Stutts J., Courtney T.K., Connor J.L. (2011). The link between fatigue and safety. Accid. Anal. Prev..

[16] May J., Baldwin C.L. (2009). Driver fatigue: the importance of identifying causal factors of fatigue when considering detection and countermeasure technologies. Transport. Res. Part F.

[17] Horrey Wj N.Y., Folkard S., Popkin S.M., Howarth H.D., Courtney T.K. (2011). Research needs and opportunities for reducing the adverse safety consequences of fatigue. Accid. Anal. Prev..

[18] Gimeno P.T., Cerezuela G.P., Montanes M.C. (2006). On the concept and measurement of driver drowsiness, fatigue, and inattention: implications for countermeasures. Int. J. Vehicle Design.

[19] Horne J.A., Reyner L.A. (1996). Counteracting driver sleepiness: effects of napping, caffeine, and placebo. Psychophysiology.

[20] Summala H., Hakkanen H., Mikkola T., Sinkkonen J. (1999). Task effects on fatigue symptoms in overnight driving. Ergonomics.

[21] Khardi S., Vallet M. (1994).

[22] Horne J.A., Reyner L.A. (1995). Sleep-related vehicle accidents. Br. Med. J..

[23] Lal S.K., Craig A. (2002). Driver fatigue: electroencephalography and psychological assessment. Psychophysiology.

[24] Liu J., Zhang C., dan Zheng C. (2010). EEG-based estimation of mental fatigue by using KPCA-HMM and complexity parameters. Biomed. Signal Process Control.

[25] Lal S.K., Craig A. (2001). A critical review of psychophysiology of driver's fatigue. Biological Physiology.

[26] Jap B.D., Lal S., dan Fischer P. (2011). Comparing Combinations of EEG activity in train drivers during monotonous driving. Expert Syst. Appl..

[27] Jagannath M. d, B V. (2014). Assessment of early onset of driver fatigue using multimodal fatigue measures in a static simulator. Appl. Ergon..

[28] Zuraida R., Iridiastadi H., Sutalaksana I.Z., Suprijanto (2019). An analysis of EEG changes during prolonged simulated driving for the assessment of driver fatigue. J. Eng. Technol. Sci..

[29] Tuncer T., Dogan S., Subasi A. (2021). EEG-based driving fatigue detection using multilevel feature extraction and iterative hybrid feature selection. Biomed. Signal Process Control.

[30] Barua S., Ahmed M.U., Ahlstrom C., Begum S. (2019). Automatic driver sleepiness detection using EEG, EOG and contextual information. Expert Syst. Appl..

[31] Zheng Y., Ma Y., Cammon J., Zhang S., Zhang J., Zhang Y. (2022). A new feature selection approach for driving fatigue EEG detection with a modified machine learning algorithm. Comput. Biol. Med..

[32] Wang H., Zhang L., Yao L. (2021). Application of genetic algorithm based support vector machine in selection of new EEG rhythms for drowsiness detection. Expert Syst. Appl..

[33] Hussein R.M., Miften F.S., George L.E. (2022). Driver drowsiness detection methods using EEG signals: a systematic review. Comput. Methods Biomech. Biomed. Eng..

[34] Dawson D., Searle A.K., Paterson J.L. (2014). Look before you (s)leep: evaluating the use of fatigue detection technologies within a fatigue risk management system for the road transport industry. Sleep Med. Rev..

[35] Liang Y., Horrey W.J., Howard M.E., Lee M.L., Anderson C., Shreeve M., O'Brien C.S., Czeisler C.A. (2019).

[36] Filtness A.J., Reyner L.A., Horne J.A. (2012). Driver sleepiness-comparisons between young and older men during a monotonous afternoon simulated drive. Biol. Psychol..

[37] Abe T., Nonomura T., Komada Y., Asaoka S., Sasai T., Ueno A., Inoue Y. (2011). Detecting deteriorated vigilance using percentage of eyelid closure time during behavioral maintenance of wakefulness tests. Int. J. Psychophysiol..

[38] Johns M.W. (1991). A new method for measuring daytime sleepiness: the Epsworth Sleepiness Scale. Sleep.

[39] Horne J.A., Ostberg O. (1976). A self-assessment questionnaire to determine morningness-eveningness in human circadian rhythms. Int. J. Chronobiol..

[40] Schleicher R., Galley N., Briest S., Galley L. (2008). Blinks and saccades as indicators of fatigue in sleepiness warnings: looking tired?. Ergonomics.

[41] Akerstedt T., Ingre M., Kecklund G., Anund A., Sandberg D., Wahde M., Phillip P., Kronberg P. (2010). Reaction to sleepiness indicator to partial sleep deprivation, time of day, and time of task in a driving simulator – the DROWSI project. J. Sleep Res..

[42] Dunbar J., Gilbert J.E., Lewis B. (2020). Exploring differences between self-report and electrophysiological indices of drowsy driving: a usability examination of a personal brain-computer interface device. J. Saf. Res..

[43] Schwilden H. (2006). Concepts of EEG processing: from power spectrum to bispectrum, fractals, entropies and all that. Best Pract. Res. Clin. Anaesthesiol..

[44] Tatum W.O. (2014).

[45] Cui J., Lan Z., Sourina O., Muller-Wittig W. (2022). EEG-based cross-subject driver drowsiness recognition with an interpretable convolutional neural network. IEEE Transact. Neural Networks Learn. Syst..

[46] Bose R., Wang H., Dragomir A., Thakor N.V., Bezerianos A., Li J. (2019). Regression-based continuous driving fatigue estimation: toward practical implementation. IEEE Transactions on Cognitive and Developmental Systems.

[47] Shi M., Yang C., Zhang D. (2021). A smart detection method of sleep quality using EEG signal and long short-term memory model. Math. Probl Eng..

[48] Xavier G., Su Ting A., Fauzan N. (2020). Exploratory study of brain waves and corresponding brain regions of fatigue on‐call doctors using quantitative electroencephalogram. J. Occup. Health.

[49] Abhang P.A., Gawali B.W., Mehrotra S.C., Abhang B.W.G.P.A., Mehrotra S.C. (2016). Introduction to EEG- and Speech-Based Emotion Recognition.

[50] Akerstedt T., Gillberg M. (1990). Subjective and objective sleepiness in the active individual. Int. J. Neurosci..

[51] De Naurois C.J., Bourdin C., Stratulat A., Diaz E., Vercher J.L. (2019). Detection and Prediction of driver drowsiness using artificial neural network models. Accid. Anal. Prev..

[52] Seo S. (2002).

[53] Wang X. d, X C. (2016). Driver drowsiness detection based on non-intrusive metrics considering individual specifics. Accid. Anal. Prev..

[54] Fu R., Wang H., Zhao W. (2016). Dynamic driver fatigue detection using hidden Markov model in real driving condition. Expert Syst. Appl..

[55] Kuhn M., Johnson K. (2019). Feature Engineering and Selection: A Practical Approach for Predictive Models.

[56] Zulfiker M.S., Kabir N., Biswas A.A., Nazneen T., Uddin M.S. (2021). An in-depth analysis of machine learning approaches to predict depression. Current Research in Behavioral Sciences.

[57] Yu L., Yu L., Yu K. (2021). A high-dimensionality-trait-driven learning paradigm for high dimensional credit classification. Financial Innov.

[58] Faul F., Erdfelder E., Buchner A., Lang A. (2009). Statistical power analyses using G*Power 3.1: tests for correlation and regression analyses. Behav. Res. Methods.

[59] Bujang M.A. (2016). Requirements for minimum sample size for sensitivity and specificity analysis. J. Clin. Diagn. Res..

[60] Subasi A. (2005). Automatic recognition of alertness level from EEG by using neural network and wavelet coefficients. Expert Syst. Appl..

[61] Perrier J., Jongen S., Vuurman E., Bocca M.L., Ramaekers J.G., Vermeeren A. (2016). Driving performance and EEG fluctuations during on-the-road driving following sleep deprivation. Biol. Psychol..

[62] Wijayanto T., Marcillia S.R., Lufityanto G., Wisnugraha B.B., Alma T.G., Abdianto R.U. (2021). The effect of situation awareness on driving performance in young sleep-deprived drivers. IATSS Res..

[63] Liang Y., Lee J.D. (2014). A hybrid Bayesian Network approach to detect driver cognitive distraction. Transport. Res. C Emerg. Technol..

[64] Roenneberg T., Wirz-Justice A., Merrow M. (2003). Life between clocks: daily temporal patterns of human chronotypes. J. Biol. Rhythm..

[65] Vapnik V.N. (1992). Principles of risk minimization for learning theory. Advances in Neural Information Processing Systems.

[66] Guyon I., Weston J., Barnhill S., Vapnik V. (2022). Gene selection for cancer classification using support vector machines. Mach. Learn..

[67] Meuleners L., Fraser M.\. (2015).

[68] Davenne D., Lericollais R., Sagaspe P., Taillard J., Gauthiera A., Espiéc S., Philip P. (2012). Reliability of simulator driving tool for evaluation of sleepiness, fatigue and driving performance. Accid. Anal. Prev..

