# Supplementary material for: Prediction of drowsiness using EEG signals in young Indonesian drivers
Source: Heliyon. 2023 Sep 3;9(9):e19499. doi: 10.1016/j.heliyon.2023.e19499 (PMC10558755; doi:10.1016/j.heliyon.2023.e19499)
Supplement: Multimedia component 1 [file mmc1.pdf]

Kuesioner Karolinska Sleepiness Scale

(*Karolinska Sleepiness Scale Questionnaire*)

Pilih salah satu nilai dari 1 sampai 9 yang paling menggambarkan kondisi kantuk Anda (*Choose one of the values from 1 to 9 that best describes your drowsiness condition*).

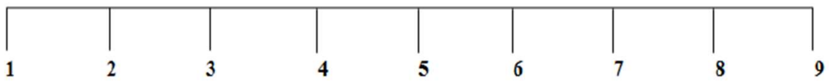

- Skala 1: Keadaan waspada penuh (*extremely alert*)
- Skala 2: Keadaan sangat waspada (*very alert*)
- Skala 3: Keadaan waspada (*alert*)
- Skala 4: Keadaan cukup waspada (*rather alert*)
- Skala 5: Antara waspada dan mengantuk (*neither alert nor sleepy*)
- Skala 6: Munculnya beberapa tanda mengantuk (*some sign of sleepiness*)
- Skala 7: Rasa mengantuk yang ringan (*sleepy, no effort to stay awake*)
- Skala 8: Rasa mengantuk yang cukup berat (*sleepy, some effort to stay awake*)
- Skala 9: Keadaan sangat mengantuk (*very sleepy, great effort to keep awake, fighting sleep*)
