# Supplementary material for: Prediction of drowsiness using EEG signals in young Indonesian drivers
Source: Heliyon. 2023 Sep 3;9(9):e19499. doi: 10.1016/j.heliyon.2023.e19499 (PMC10558755; doi:10.1016/j.heliyon.2023.e19499)
Supplement: Multimedia component 2 [file mmc2.pdf]

Personal Data Questionnaire

Prediction of Drowsiness using EEG signal in Young Indonesian Drivers

Please fill out the following questions and consent form in the next pages.

Name	:	
Gender	:	M / F
Phone Number	:	
Date of Birth	:	
Age	:	years old
Body Temperature	:	°C
Weight	:	kg
Height	:	cm
Previous Bedtime	:	(fill out by researcher)
Blood Pressure	:	(fill out by researcher)

CONSENT FORM
Prediction of Drowsiness Using EEG Signal in Young Indonesian Drivers

You are willing to participate in research conducted by researcher (Dr. Maya Arlini Puspasari) from the University of Indonesia because you meet the following requirements:

- Aged 17-35 years
- Already have a driver's license and get used to driving for at least one year
- Having normal eyes or normal eye correction
- Being in good health condition
- Having normal sleep durations (6 h/day on average) and the absence of sleep disturbance
- Consume caffeine (tea or coffee) less than or equal to 1 cup daily
- Smoke less than or equal to 1 cigarette/day
- Not taking medicines

Your participation in this research is based on your willingness and consent. Please read the information written below carefully. Please ask if there is any information that is not understood.

RESEARCH OBJECTIVES
- Determine the best parameters on the EEG signal indicator to detect fatigue
- Designing EEG signal-based fatigue indicators on driving tasks

RESEARCH PROCEDURE
In the series of studies there are 10 sequences of procedures, each of which is described as follows:
1. Data collection is carried out one by one participant
2. Participants must take adequate sleep at least 7 hours or less than 5 hours (depending on the combination) before data collection and are not allowed to consume caffeine and other medicines for 24 hours before data collection
3. Participants will be directed to fill in personal data questionnaires and consent form to ensure Participants' participation in this study
4. Participants take weight and blood pressure measurements
5. Participants will be given brief instructions regarding the data retrieval flow and instructions given to the respondent only to drive properly and correctly during data retrieval
6. Researchers install EEG tools on participants
7. Researchers will ask about participants' sleepiness levels at that time based on the Karolinska Sleepiness Scale (KSS)
8. Participants will do driving activities on the driving simulator for 1 hour
9. Before driving session, participants were given lunch by researcher
10. If you experience problems, you are allowed if you wish to end the driving session faster than you should or do not continue to participate in this study

DRIVING TASKS
In this experiment you are required to drive using a driving simulator. When driving, you are required to:

- Maintain safety, comfort, and driving safety for the driver
- Do not take caffeine and other drugs for 24 hours before data collection
- Commit to come at the agreed meeting time with the researcher
- Drive at a reasonable speed just like when driving on a real highway
- Sit in a reasonable position while on driving duty
- Does not activate mobile phone
- Not going to the restroom at the time of the experiment. You are allowed to go at the time the time before the experiment and at the time of rest
- It is not allowed to build intensive communication (chatting) with researchers during the experiment
- Be as cooperative and reasonable as you normally would drive
- Report complaints and your ability limits
- Before driving, you are expected to come to the laboratory early for driving practice in the simulator as well as weight and blood pressure measurements.

DATA COLLECTION

This session is conducted during the performance of driving tasks. In each data retrieval session, you are required to undergo a series of data retrieval procedures as follows:

- EEG: A wireless electroencephalography (EEG) device will be installed in your head during a driving session (1 hour). The device contains 5 electrodes that are given electrolyte fluid. The tool records your brainwave data with the Emotiv Insight app.
- KSS: Assess the condition of yourself by choosing one of the 9 condition statements that you feel best suits your condition when asked. This process is executed before, during, and after you perform a driving task.
- Camera: The camera records the condition of the cabin and your facial expression during the tasks.

In addition to the data collection carried out during the driving session, there are other data collections that were carried out before the experiment started, namely:

- Personal Data Questionnaire
- Weight Measurement
- Blood Pressure Measurement

Rest

This session is conducted at the end of the driving session after 1 hour. One break session is run within ± 15 minutes. During that time, you are allowed to rest using the facilities provided.

RISKS

With the use of simulators in this study, there is no risk posed by your participation in this research. After completing the entire activity in the study, you may experience drowsiness and fatigue. Researchers also advise and allow you to take a short break at the facilities provided at the resting period.

ADVANTAGE

As a participant, you will get several benefits as follows:

- Providing a valuable major contribution to the research in the field of transportation safety
- Get consumption in the form of lunch
- Get a gopay balance of IDR 50,000

PRIVACY

Data about your identity (name, age, etc.) will be kept confidential. The data used and may be published in the scientific community is data related to the results of experiment.

CONTACT INFORMATION

If you have any questions and other things you want to convey regarding this research, please contact:

Name : Dr. Maya Arlini Puspasari

Office : Ergonomics Centre Laboratory, Department of Industrial Engineering, Faculty of Engineering, University of Indonesia, Depok 16424.

Phone : 021-78888805 / 085776008824

PARTICIPANT SIGNATURE

I, the undersigned have read and understood the above information. I approve of my participation in this study. I promise to follow this research earnestly, honestly, and openly and adhere to what is required by the researcher during this study and not tell the details of the research to unauthorized parties.

____

Name

______________________________________ ________________

Signature Date

RESEARCHER SIGNATURE

As a researcher, I have provided and explained the information needed by participants to take part in this study. I have submitted that the participation of participants in the research is based on the consent and willingness of the person concerned.

____

Name: Dr. Maya Arlini Puspasari

____

Signature Date
