# Supplementary material for: Prediction of drowsiness using EEG signals in young Indonesian drivers
Source: Heliyon. 2023 Sep 3;9(9):e19499. doi: 10.1016/j.heliyon.2023.e19499 (PMC10558755; doi:10.1016/j.heliyon.2023.e19499)
Supplement: Multimedia component 3 [file mmc3.pdf]

KUESIONER MORNINGNESS EVENINGNESS (*MORNINGNESS AND EVENINGNESS QUESTIONNAIRE*)

Adopted from Horne dan Ostberg, 1976

Nama (*name*): Tanggal (*date*):

Untuk setiap pertanyaan, berikan jawaban yang paling mencerminkan kebiasaan anda selama beberapa minggu terakhir (*For each question, please select the answer that best describes you by circling the point value that best indicates how you have felt in recent weeks*)

1. Jam berapa anda bangun tidur ketika waktu pagi bebas (tidak ada kegiatan)? (*Approximately what time would you get up if you were entirely free to plan your day?*)
   [5] 05.00 – 06.30 (5 – 6.30 AM)
   [4] 06.30 – 07.45 (6.30 – 7.45 AM)
   [3] 07.45 – 09.45 (7.45 – 9.45 AM)
   [2] 09.45 – 11.00 (9.45 – 11 AM)
   [1] 11.00 – 12.00 (11 AM – 12 PM)
2. Jam berapa anda tidur ketika waktu malam anda (tidak ada kegiatan)? (*Approximately what time would you go to bed if you were entirely free to plan your evening?*)
   [5] 20.00 – 21.00 (8 – 9 PM)
   [4] 21.00 – 22.15 (9 – 10.15 PM)
   [3] 22.15 – 00.30 (10.15 PM – 12.30 AM)
   [2] 00.30 – 01.45 (12.30 – 1.45 AM)
   [1] 01.45 – 03.00 (1.45 – 3 AM)
3. Jika anda terbiasa bangun tidur pada waktu yang sudah teratur, seberapa besar ketergantungan anda pada jam weker? (*If you usually have to get up at a specific time in the morning, how much do you depend on an alarm clock?*)
   [4] Tidak sama sekali (*Not at all*)
   [3] Sedikit (*Slightly*)
   [2] Cukup (*Somewhat*)
   [1] Sangat (*Very much*)
4. Seberapa mudah anda bangun pagi (ketika anda tidak terbangun tiba-tiba)? (*How easy do you find it to get up in the morning (when you are not awakened unexpectedly)?*)
   [1] Sangat sulit (*Very difficult*)
   [2] Agak sulit (*Somewhat difficult*)
   [3] Cukup mudah (*Fairly easy*)
   [4] Sangat mudah (*Very easy*)
5. Seberapa terjaga anda dalam periode setengah jam pertama setelah terbangun di pagi hari? (*How alert do you feel during the first half hour after you wake up in the morning?*)
   [1] Sama sekali tidak terjaga (*Not at all alert*)
   [2] Sedikit terjaga (*Slightly alert*)
   [3] Cukup terjaga (*Fairly alert*)
   [4] Sangat terjaga (*Very alert*)
6. Seberapa lapar anda dalam periode setengah jam pertama setelah terbangun? (*How hungry do you feel during the first half hour after you wake up?*)
   [1] Sama sekali tidak lapar (*Not at all hungry*)
   [2] Sedikit lapar (*Slightly hungry*)
   [3] Cukup lapar (*Fairly hungry*)

[4] Sangat lapar (*Very hungry*)

7. Dalam periode setengah jam setelah anda bangun di pagi hari, bagaimana perasaan anda? (*During the first half hour after you wake up in the morning, how do you feel?)*
   [1] Sangat lelah (*Very tired*)
   [2] Cukup lelah (*Fairly tired*)
   [3] Cukup segar (*Fairly refreshed*)
   [4] Sangat segar (*Very refreshed*)
8. Ketika anda tidak memiliki kegiatan di hari berikutnya, bagaimana waktu tidur anda dibandingkan kebiasaan tidur anda? (*If you had no commitments the next day, what time would you go to bed compared to your usual bedtime?*)
   [4] Sangat jarang atau tidak pernah terlambat (*Seldom or never later*)
   [3] Terlambat kurang dari 1 jam (*Less than 1 hour later*)
   [2] Terlambat 1-2 jam (*1-2 hours later*)
   [1] Terlambat lebih dari 2 jam (*More than 2 hours later*)
9. Anda berjanji untuk mengikuti olahraga dengan teman anda, yang akan dilaksanakan selama 1 jam selama dua kali seminggu pada pukul 07.00-08.00. Dengan memperhatikan jam biologis anda, bagaimana performa anda? (*You have decided to do physical exercise. A friend suggests that you do this for one hour twice a week, and the best time for him is between 7-8 AM (07-08 h). Bearing in mind nothing but your own internal "clock," how do you think you would perform?*)
   [4] Dalam kondisi yang prima (*Would be in good form*)
   [3] Dalam kondisi yang baik (*Would be in reasonable form*)
   [2] Sulit dalam mengikuti (*Would find it difficult*)
   [1] Sangat sulit dalam mengikuti (*Would find it very difficult*)
10. Pada jam berapa di malam hari anda merasa lelah dan butuh untuk tidur? (*At approximately what time in the evening do you feel tired, and, as a result, in need of sleep?*)
    [5] 20.00 – 21.00 (8 – 9 PM)
    [4] 21.00 – 22.15 (9 – 10.15 PM)
    [3] 22.15 – 00.45 (10.15 PM – 12.45 AM)
    [2] 00.45 – 02.00 (12.45 – 2 AM)
    [1] 02.00 – 03.00 (2 – 3 AM)
11. Anda ingin berada pada kondisi prima untuk menghadapi tes yang melelahkan yang berdurasi 2 jam. Anda bebas menentukan waktu tes tersebut. Dengan mempertimbangkan jam biologis anda, di jam berapakah anda akan mengambil tes tersebut? (*You want to be at your peak performance for a test that you know is going to be mentally exhausting and will last two hours. You are entirely free to plan your day. Considering only your "internal clock," which one of the four testing times would you choose?*)
    [6] 08.00 – 10.00 (8 – 10 AM)
    [4] 11.00 – 13.00 (11 AM – 1 PM)
    [2] 15.00 – 17.00 (3 – 5 PM)
    [0] 19.00 – 21.00 (7 – 9 PM)
12. Jika anda tidur jam 23.00, seberapa lelah anda? (*If you got into bed at 11 PM (23 h), how tired would you be?*)
    [0] Sama sekali tidak lelah (*Not at all tired*)
    [2] Sedikit lelah (*A little tired*)
    [3] Cukup lelah (*Fairly tired*)
    [5] Sangat lelah (*Very tired*)

13. Anda tidur beberapa jam lebih terlambat daripada biasanya, tapi di hari berikutnya tidak harus bangun pagi. Apakah yang akan anda lakukan esok harinya? (*For some reason you have gone to bed several hours later than usual, but there is no need to get up at any particular time the next morning. Which one of the following are you most likely to do?*)
    [4] Akan terbangun pada waktu biasa, dan tidak akan tidur kembali (*Will wake up at usual time, but will not fall back asleep*)
    [3] Akan terbangun pada waktu biasa dan mengantuk (*Will wake up at usual time and will doze thereafter*)
    [2] Akan terbangun pada waktu biasa, namun akan tertidur kembali (*Will wake up at usual time, but will fall asleep again*)
    [1] tidak akan terbangun pada waktu biasanya (lebih lambat daripada biasanya) (*Will not wake up until later than usual*)
14. Anda diharuskan berjaga pada pukul 04.00-06.00 pagi dan tidak ada kegiatan pada hari berikutnya. Pilihan apa yang sesuai dengan anda? (*One night you have to remain awake between 4-6 AM in order to carry out a night watch. You have no time commitments the next day. Which one of the alternatives would suit you best?*)
    [1] Tidak akan tidur sampai penjagaan berakhir (*Would not go to bed until the watch is over*)
    [2] Tidur siang dan akan tidur setelah penjagaan berakhir (*Would take a nap before and sleep after*)
    [3] Tidur cukup sebelum penjagaan dan akan tidur siang setelahnya (*Would take a good sleep before and nap after*)
    [4] Akan tidur hanya sebelum penjagaan (*Would sleep only before the watch*)
15. Anda memiliki waktu 2 jam untuk kegiatan fisik yang berat. Anda bebas menentukan waktu kegiatan tersebut. Dengan mempertimbangkan jam biologis anda, di jam berapakah anda akan mengambil tes tersebut? (*You have two hours of hard physical work. You are entirely free to plan your day. Considering only your internal "clock," which of the following times would you choose?*)
    [4] 08.00 – 10.00
    [3] 11.00 – 13.00
    [2] 15.00 – 17.00
    [1] 19.00 – 21.00
16. Anda berjanji untuk mengikuti olahraga dengan teman anda, yang akan dilaksanakan selama 1 jam selama dua kali seminggu pada pukul 22.00-23.00. Dengan memperhatikan jam biologis anda, bagaimana performa anda? (*You have decided to do physical exercise. A friend suggests that you do this for one hour twice a week. The best time for her is between 10-11 PM. Bearing in mind only your internal "clock," how well do you think you would perform?*)
    [1] Dalam kondisi yang prima (*Would be in good form*)
    [2] Dalam kondisi yang baik (*Would be in reasonable form*)
    [3] Sulit dalam mengikuti (*Would find it difficult*)
    [4] Sangat sulit dalam mengikuti (*Would find it very difficult*)
17. Asumsikan bahwa anda dapat memilih jam kerja anda, yang berdurasi selama 5 jam, pekerjaannya menarik, dan anda akan dibayar berdasarkan performa anda. Pada waktu kapan anda memulai pekerjaan tersebut? (*Suppose you can choose your own work hours. Assume that you work a five-hour day (including breaks), your job is interesting, and you are paid based on your performance. At approximately what time would you choose to begin?*)
    [5] 5 jam mulai dari pukul 05.00 – 08.00 (*5 hours starting between 5–8 AM*)
    [4] 5 jam mulai dari pukul 08.00 – 09.00 (*5 hours starting between 8–9 AM*)
    [3] 5 jam mulai dari pukul 09.00 – 14.00 (*5 hours starting between 9 AM–2 PM*)

[2] 5 jam mulai dari pukul 14.00 – 17.00 (*5 hours starting between 2–5 PM*)
[1] 5 jam mulai dari pukul 17.00 – 04.00 (*5 hours starting between 5 PM–4 AM*)

18. Pada waktu kapan anda merasakan kondisi paling prima? (*At approximately what time of day do you usually feel your best?*)
    [5] 05.00 – 08.00 (5 – 8 AM)
    [4] 08.00 – 10.00 (8 – 10 AM)
    [3] 10.00 – 17.00 (10 AM – 5 PM)
    [2] 17.00 – 22.00 (5 – 10 PM)
    [1] 22.00 – 05.00 (10 PM – 5 AM)
19. Menurut anda, anda berada dalam kategori yang mana? (*One hears about "morning types" and"evening types." Which one of these types do you consider yourself to be?*)
    [6] Dalam kategori "tipe pagi (*morning type*)" (*Definitely a morning type*)
    [4] Lebih "tipe pagi (*morning type*)" daripada "tipe malam (*evening type*)" (*Rather more a morning type than an evening type*)
    [2] Lebih "tipe malam (*evening type*)" daripada "tipe pagi (*morning type*)" (*Rather more an evening type than a morning type*)
    [1] Dalam kategori "tipe malam (*evening type*)" (*Definitely an evening type*)
